# Adaptive Neuro-Fuzzy Inference System Applied QSAR with Quantum Chemical Descriptors for Predicting Radical Scavenging Activities of Carotenoids

**DOI:** 10.1371/journal.pone.0140154

**Published:** 2015-10-16

**Authors:** Changho Jhin, Keum Taek Hwang

**Affiliations:** Department of Food and Nutrition, Research Institute of Human Ecology, Seoul National University, Seoul, Korea; Center for Nanosciences and Nanotechnology, MEXICO

## Abstract

One of the physiological characteristics of carotenoids is their radical scavenging activity. In this study, the relationship between radical scavenging activities and quantum chemical descriptors of carotenoids was determined. Adaptive neuro-fuzzy inference system (ANFIS) applied quantitative structure-activity relationship models (QSAR) were also developed for predicting and comparing radical scavenging activities of carotenoids. Semi-empirical PM6 and PM7 quantum chemical calculations were done by MOPAC. Ionisation energies of neutral and monovalent cationic carotenoids and the product of chemical potentials of neutral and monovalent cationic carotenoids were significantly correlated with the radical scavenging activities, and consequently these descriptors were used as independent variables for the QSAR study. The ANFIS applied QSAR models were developed with two triangular-shaped input membership functions made for each of the independent variables and optimised by a backpropagation method. High prediction efficiencies were achieved by the ANFIS applied QSAR. The R-square values of the developed QSAR models with the variables calculated by PM6 and PM7 methods were 0.921 and 0.902, respectively. The results of this study demonstrated reliabilities of the selected quantum chemical descriptors and the significance of QSAR models.

## Introduction

Carotenoids are natural pigments that have various physiological activities such as conversion to vitamin A and immunological activities [1–3]. One of the important physiological activities of carotenoids is their radical scavenging activity [4–7]. The radical scavenging activity of carotenoids, mainly contributed by conjugated polyene structures of carotenoids [8], is related with direct quenching of singlet oxygen and radical species [9,10]. On the other hand, quantitative structure-activity relationship (QSAR) studies regarding antioxidant activities and radical scavenging activities of natural products have been conducted to understand radical scavenging mechanisms and to predict the activities [11–13]. The quantum chemical descriptors (such as ionisation energy, electrophilicity, highest occupied molecular orbital (HOMO) and lowest unoccupied molecular orbital (LUMO) energies, etc.) have been used as independent variables in QSAR studies, because the quantum chemical descriptors characterise electronic and geometric properties of molecules which affect activities of the molecules [14]. There have been QSAR studies on antioxidant activities of carotenoids [15,16]; however, correlation between various quantum chemical descriptors and antioxidant activities of carotenoids have not been reported. Since quantum chemical descriptors represent physicochemical properties of molecules, QSAR models for antioxidant activities of carotenoids could be developed by using their quantum chemical descriptors.

The relationship between radical scavenging activity and quantum chemical descriptors was studied previously [12,14,17–19]. The relationship between quantum chemical descriptors and the radical scavenging activities can be explained theoretically. Radical species could be scavenged by single electron transfer from a radical scavenger [20]. In a physicochemical manner, it is considered that compounds with low ionisation energy tend to be good radical scavengers because electron transfer from the HOMO of a radical scavenger to the single occupied molecular orbital (SOMO) of a radical is occurred more easily on the compounds with a lower ionisation energy compared to the compounds with a higher ionisation energy [14]. In case of nucleophilic radical species, the radical scavenging reaction is attributed to the interaction between SOMO of a radical and the LUMO of a radical scavenger [21,22]. According to Koopmans’ theorem [23], the energy level of LUMO is a negative electron affinity; therefore, a compound with a higher electron affinity tends to be attacked easily by a nucleophilic radical. The energy level of the SOMO of radical is dropped by interaction with the LUMO of a radical scavenger, followed by single electron transfer from HOMO of radical scavenger to the SOMO of radical to scavenge it. The low HOMO-LUMO gap allows electron transfer from HOMO more easily. Since the concept of chemical hardness is based on the HOMO-LUMO gap [24], compounds with low chemical hardness tend to have high radical scavenging activity [13,14]. In addition, the driving force for electron transfer is chemical potential (negative electronegativity), which is the slope of the energy versus number of electron curve [25]. In this manner, the chemical potential indicates the rate and the direction of chemical reactions including the radical scavenging activity.

Linear regression method is usually applied to develop QSAR models in several studies [13,15–17,26]; however, the interaction effect between independent variables and nonlinear relationships could not be interpreted easily by the linear regression method. Adaptive neuro-fuzzy inference system (ANFIS) is an artificial neural network (ANN) applied fuzzy inference system. ANFIS is an empirical method that defines rules by training and backpropagation processes, and multivariate nonlinear relationship could be solved by fuzzy if-then rules [27]. Thus, in this study, ANFIS applied QSAR models were developed with quantum chemical descriptors for predicting and analysing antioxidant activities of carotenoids.

## Materials and Methods

### Data set

**Fig 1:**
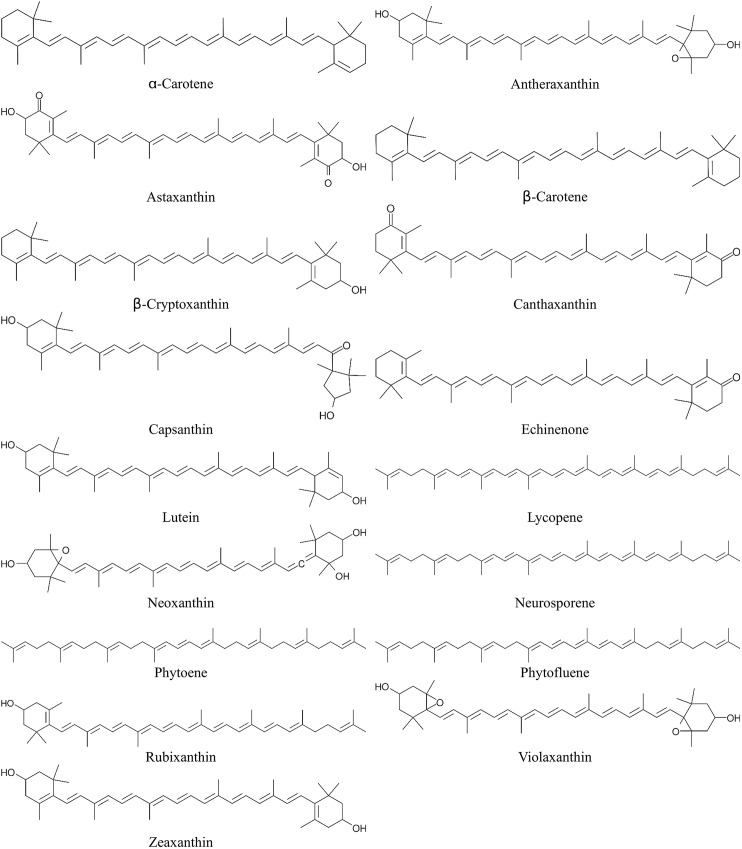
Carotenoid structures presented by Müller et al. [4].

Total three data sets of radical scavenging activities of carotenoids were adapted from previous studies. The first trolox equivalent antioxidant capacity(TEAC) data set by Müller et al. [4] was constituted of 17 carotenoids (Fig 1), and the second data set by Miller et al. [5] was constituted of 8 carotenoids. A DPPH radical scavenging activity data set was adapted from the report by Jiménez-Escrig et al. [28].

### Molecular structure preparation

The structures of all-trans α-carotene, antheraxanthin, astaxanthin, β-carotene, β-cryptoxanthin, canthaxanthin, capsanthin, echinenone, lutein, lycopene, neoxanthin, neurosporene, phytoene, phytofluene, rubixanthin, violaxanthin and zeaxanthin were obtained from ZINC database [29] as mol2 format and converted into MOPAC input format by Openbabel [30].

### Quantum chemical calculations

Semi-empirical PM6 [31] and PM7 [32] quantum chemical calculations were done by MOPAC2012 [33]. The heat of formation of structurally optimised carotenoid molecules was retrieved from MOPAC output files. The quantum chemical properties of ionisation energy (***I***), electron affinity (***A***), chemical hardness (***η***), electronegativity (***χ***), chemical potential (***μ***) and electrophilicity (***ω***) were calculated as following equations:

I=E(N−1)−E(N) (1)

A=E(N)−E(N+1) (2)

η=(I−A)/2 (3)

χ=−μ=(I+A)/2 (4)

$$\omega =\mu^{2}/2\eta$$ (5)

where ***E*(*N*)** is energy of neutral carotenoid molecule, ***E*(*N* – 1)** is energy of monovalent carotenoid cation, and ***E*(*N* + 1)** is energy of monovalent anion.

Likewise of the quantum chemical properties of neutral carotenoid molecules, the ionisation energy (***I***_***cat***_), electron affinity (***A***_***cat***_), chemical hardness (***η***_***cat***_), electronegativity (***χ***_***cat***_), chemical potential (***μ***_***cat***_) and electrophilicity (***ω***_***cat***_) of monovalent cationic carotenoid molecules were calculated. To analyse cross-level effects between neutral and cationic carotenoid molecules, the cross-product terms of quantum chemical property values of neutral carotenoid molecules and those of cationic carotenoid molecules were calculated and abbreviated as ionisation energy (***I***_***cross***_), electron affinity (***A***_***cross***_), chemical hardness (***η***_***cross***_), electronegativity (***χ***_***cross***_), chemical potential (***μ***_***cross***_) and electrophilicity (***ω***_***cross***_).

### Quantitative structure-activity relationship

#### Correlation analysis

Prior to developing QSAR models, Pearson’s correlation analysis between quantum chemical properties and antioxidant activities of carotenoids was done by using GNU R (http://cran.r-project.org/). Quantum chemical properties highly correlated with antioxidant activities were selected as independent variables for QSAR models.

#### Adaptive neuro-fuzzy inference system

In order to develop QSAR models, ANFIS was applied using Matlab 8.2 (Mathworks, Natick, MA, USA). From the correlation analysis, ***I***, ***I***_***cat***_ and ***μ***_***cross***_ were chosen as independent variables for QSAR models. Each independent variable was standardised to be adjusted to the same scale by following equation:

xistd=xi−x¯∑i=1N(xi−x¯)2N (6)

where $x_{i}^{std}$ is standardised value of the sample, ***x***_***i***_ is original value of the sample, **$\bar{x}$** is the mean of each variable, and ***N*** is the number of the samples in the data set.

**Fig 2:**
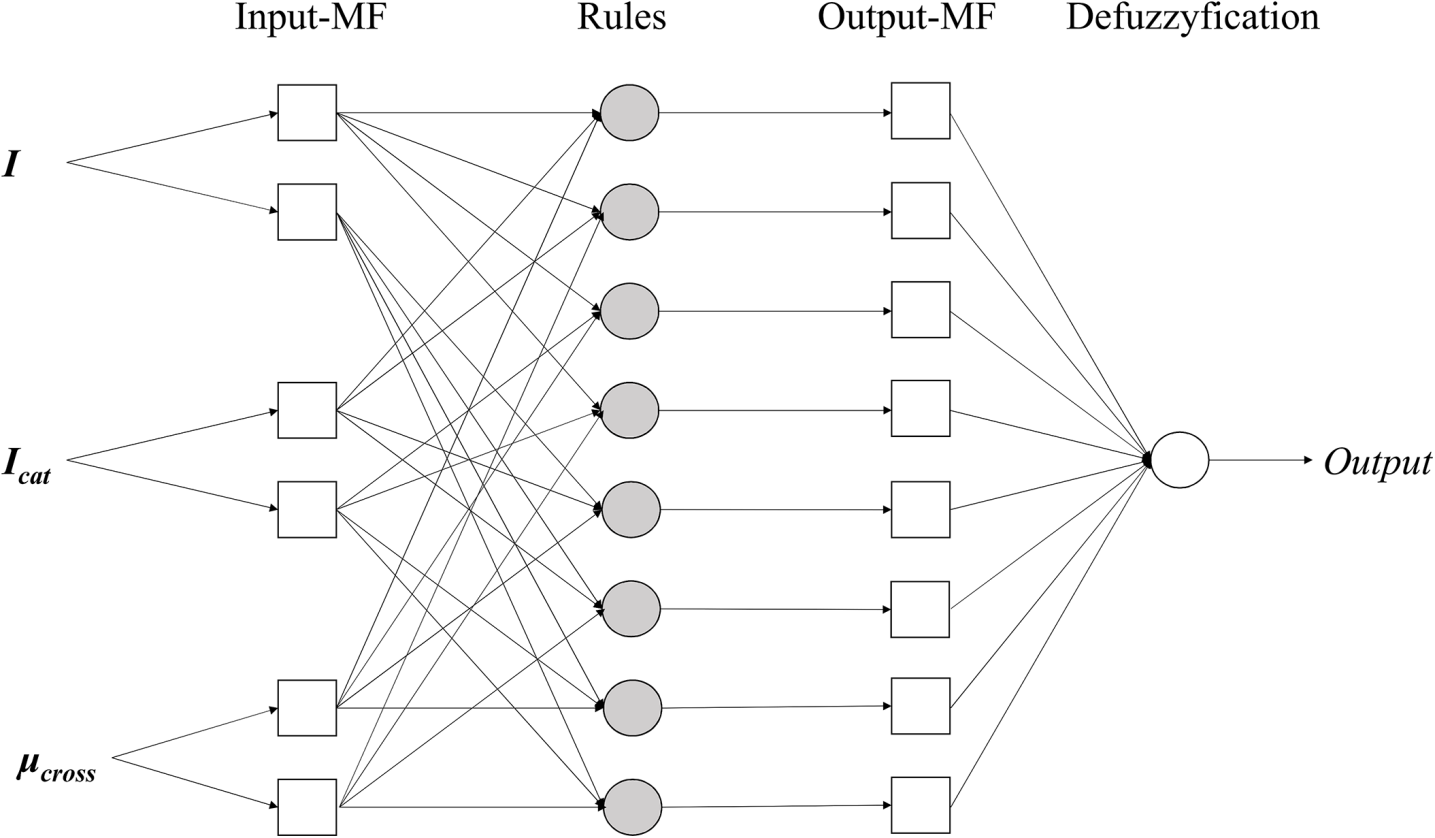
Developed adaptive neuro-fuzzy inference system (ANFIS) structure.

Two triangular-shaped membership functions were applied for each independent variable, 8 if-then rules and 8 linear type output functions were applied for ANFIS. To train and optimise ANFIS models, back-propagation method was used. The structure of ANFIS is illustrated on Fig 2. To validate constructed QSAR models, 1000 times of bootstrap resampling validation procedure were applied. To measure prediction efficiency, mean absolute error (MAE) was calculated as follows:

$$\mathrm{MAE}=\frac{1}{N}\sum_{i=1}^{n}\left|y_{i}^{\prime }-y_{i}\right|$$ (7)

where $y_{i}^{\prime }$ is predicted value resulted from QSAR model and ***y***_***i***_ is experimental value from the literature.

## Results and Discussion

### Correlation analysis

The molecular structures and the list of carotenoids adapted from the study of Müller et al. [4] are illustrated in Fig 1, and their calculated quantum chemical properties by PM6 method are presented in Table 1. The quantum chemical descriptors of monovalent cationic carotenoid molecules were also calculated because both of the neutral and monovalent cationic carotenoid molecules exist at the same time during the radical scavenging reaction [34,35]. In addition, the cross-product terms should be calculated and introduced including interaction effects between neutral and cationic carotenoid molecules on QSAR models.

**Table 1:** Molecular quantum chemical properties of carotenoids calculated by PM6 semi-empirical quantum chemistry method (kcal/mol).

	*I* ^a^	*A*	*η*	*μ*	*χ*	*ω*	*I*_*cat*_ ^b^	*A*_*cat*_	*η*_*cat*_	*μ*_*cat*_	*χ*_*cat*_	*ω*_*cat*_
**α-Carotene**	142.76	49.83	46.46	-96.29	96.29	99.78	190.12	142.76	23.68	-166.44	166.44	584.83
**Antheraxanthin**	145.03	51.84	46.59	-98.44	98.44	103.98	194.18	145.03	24.57	-169.60	169.60	585.35
**Astaxanthin**	151.37	60.29	45.54	-105.83	105.83	122.98	203.93	151.37	26.28	-177.65	177.65	600.41
**β-Carotene**	142.96	49.00	46.98	-95.98	95.98	98.05	185.09	142.96	21.06	-164.02	164.02	638.68
**β-Cryptoxanthin**	142.91	50.20	46.35	-96.55	96.55	100.57	187.17	142.91	22.13	-165.04	165.04	615.32
**Canthaxanthin**	148.87	55.19	46.84	-102.03	102.03	111.12	199.23	148.87	25.18	-174.05	174.05	601.49
**Capsanthin**	146.36	62.30	42.03	-104.33	104.33	129.50	199.33	146.36	26.49	-172.84	172.84	563.97
**Echinenone**	143.88	53.38	45.25	-98.63	98.63	107.50	193.72	143.88	24.92	-168.80	168.80	571.77
**Lutein**	144.74	55.52	44.61	-100.13	100.13	112.38	192.37	144.74	23.81	-168.55	168.55	596.61
**Lycopene**	139.28	53.45	42.92	-96.36	96.36	108.19	183.63	139.28	22.18	-161.46	161.46	587.78
**Neoxanthin**	145.50	54.32	45.59	-99.91	99.91	109.47	197.76	145.50	26.13	-171.63	171.63	563.63
**Neurosporene**	142.19	48.31	46.94	-95.25	95.25	96.64	192.75	142.19	25.28	-167.47	167.47	554.72
**Phytoene**	160.00	19.10	70.45	-89.55	89.55	56.92	248.57	160.00	44.29	-204.28	204.28	471.15
**Phytofluene**	151.72	33.62	59.05	-92.67	92.67	72.71	222.44	151.72	35.36	-187.08	187.08	494.86
**Rubixanthin**	140.67	52.91	43.88	-96.79	96.79	106.75	187.20	140.67	23.26	-163.94	163.94	577.59
**Violaxanthin**	147.79	52.65	47.57	-100.22	100.22	105.56	199.75	147.79	25.98	-173.77	173.77	581.09
**Zeaxanthin**	144.51	51.03	46.74	-97.77	97.77	102.26	188.52	144.51	22.01	-166.51	166.51	629.94

^a^ ***I*, *A*, *η*, *μ*, *χ*,** and ***ω*** are ionisation energy, electron affinity, chemical hardness, chemical potential, electronegativity, and electrophilicity of neutral carotenoid, respectively.

^b^ ***I***_***cat***_, ***A***_***cat***_, ***η***_***cat***_ **, *μ***_***cat***_ **, *χ***_***cat***_, and ***ω***_***cat***_ are ionisation energy, electron affinity, chemical hardness, chemical potential, electronegativity, and electrophilicity of cationic carotenoid, respectively.

Pearson’s correlation coefficients between TEAC values of carotenoids from Müller et al. [4] and quantum chemical properties calculated by PM6 and PM7 methods are presented in Table 2. The correlations analysed by both of the PM6 and PM7 methods showed identical tendencies. The negative correlation between the TEAC and calculated ***I*** value could be explained in physicochemical manner. Due to conjugation of double bonds on the long carbon chain skeleton of carotenoids, carotenoids oxidise easily via electron transfer mechanism [6–8,36]. Since electron transfer is a main mechanism of antioxidant activity of carotenoids, carotenoids with lower ***I*** are expected to have higher antioxidant capacity compared to carotenoids with higher ***I***. Not only the ***I***, but also the ***I***_***cat***_ had a significant correlation with the TEAC. This result postulates a radical scavenging mechanism of carotenoids that two electrons of a single carotenoid molecule transfer to radical species to scavenge them. Previous studies also reported that carotenoids undergo sequential loss of two electrons in oxidation reaction [34,35,37]. For the reason that the ***I*** was lower than the ***I***_***cat***_ (Table 1) and the ***I*** was more significantly correlated with the TEAC compared to the ***I***_***cat***_ (Table 2), electron transfer process from neutral carotenoid molecule to radical occurs more favourably than that from monovalent carotenoid cation.

**Table 2:** Pearson’s correlation coefficients between radical scavenging activities and quantum chemical descriptors of carotenoids calculated by PM6 and PM7 methods.

	PM6			PM7		
	Neutral	Cation	Product	Neutral	Cation	Product
**Ionisation energy**	-0.785***	-0.653**	-0.683**	-0.732***	-0.632**	-0.650**
**Electron affinity**	0.211	-0.785***	0.099	0.245	-0.732***	0.150
**Chemical hardness**	-0.440	-0.572*	-0.493*	-0.434	-0.581*	-0.501*
**Chemical potential**	0.226	0.692**	0.847***	0.143	0.659**	0.830***
**Electronegativity**	-0.226	-0.692**	-0.847***	-0.143	-0.659**	-0.830***
**Electrophilicity**	0.148	0.416	0.226	0.200	0.570*	0.346

*p < 0.05

**p < 0.01

***p < 0.001

Also, a significantly high correlation between ***μ***_***cross***_ (-***χ***_***cross***_) and TEAC values (p<0.001) could be noted, but lower significances were observed in the cases of ***μ*** and ***μ***_***cation***_ (Table 2). This result postulates that antioxidant activities of carotenoids were mainly contributed by both of the neutral and cationic carotenoid molecules rather than either of them. Since the chemical potential (negative electronegativity) is a thermodynamic property derived by differentiating the energy with respect to the number of electrons [25], it indicates the direction of chemical reactions as a partial free energy. Thus, a positive relationship was observed between the chemical potential and the TEAC. The postulated mechanism of radical scavenging activities of carotenoids with limited information from correlation analysis is related with the sequential donation of two electrons to radical species. Chemical potential, ***μ***, as a driving force for electron transfer [25] is responsible for first electron transfer reaction and ***μ***_***cat***_ is responsible for the second electron transfer reaction to radical species. The electron transfer occurs not only between the carotenoids and radical species, but also between the neutral and cationic carotenoids [35,38]. The significant relationship between ***μ***_***cross***_ and the TEAC was supposed to be caused by the interaction effect between the neutral and cationic carotenoids. In this physicochemical manner, the quantum chemical descriptor of chemical potential could be applied on QSAR studies. Worachartcheewan et al. [11] introduced chemical potential to predict radical scavenging activities of curcuminoids, and Pasha et al. [17] developed QSAR model for radical scavenging activities of flavonoids with electronegativity as a dependent variable.

**Fig 3:**
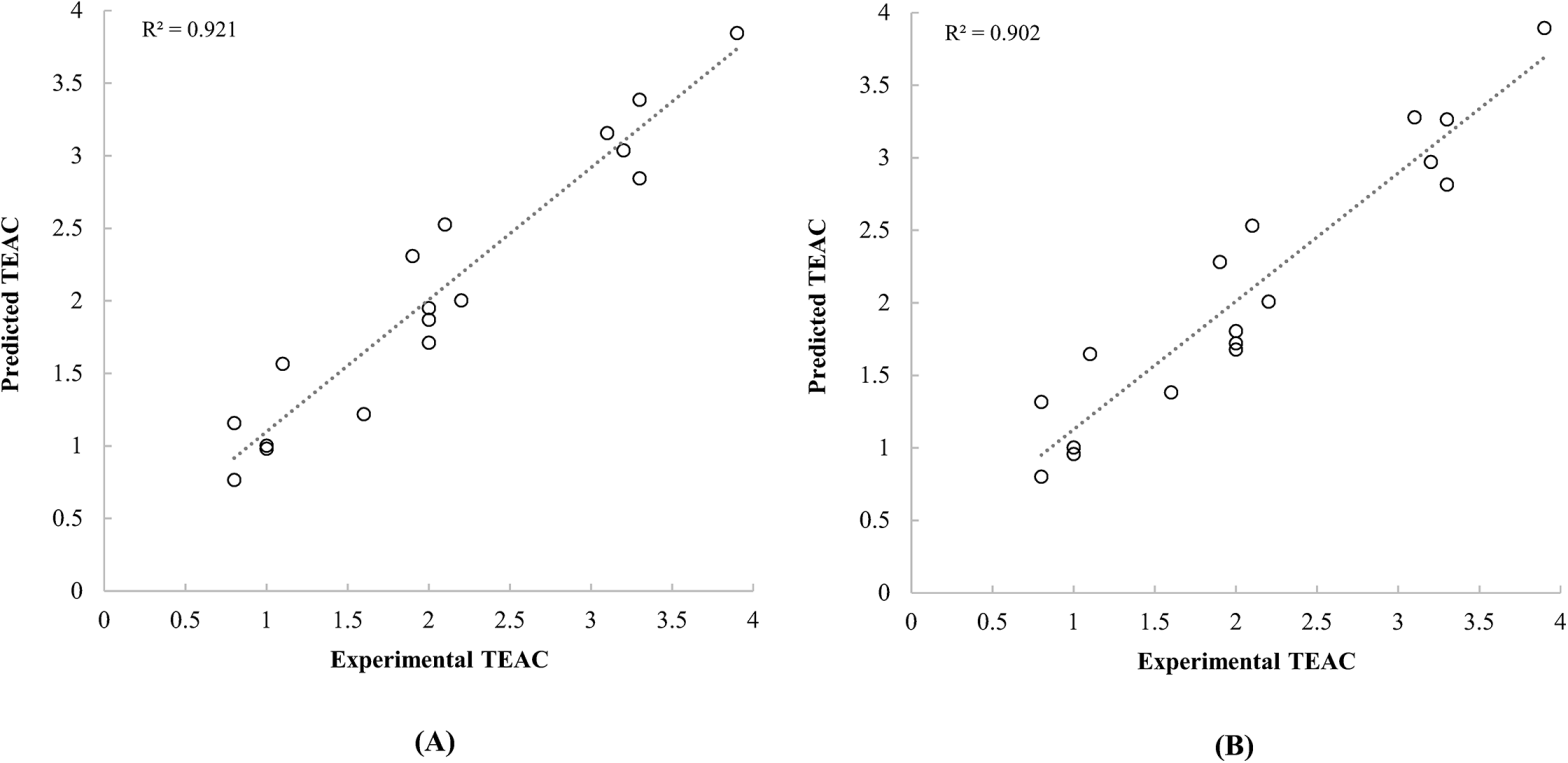
Scatter plot between the experimental TEAC of carotenoids reported by Müller et al. [4] and the predicted TEAC based on the quantum chemical descriptors calculated by PM6 (A) and PM7 (B) methods.

From the result of correlation analysis, ***I***, ***I***_***cat***_, and ***μ***_***cross***_ were selected as dependent variables for developing QSAR models, for reason that these properties had a significant correlation with antioxidant activities of carotenoids. Most of previous QSAR studies developed the prediction models by linear regression [13,15,17,18,26]. However, traditional QSAR models by linear regression analysis ignore the interaction effects between dependent variables, thus this ignorance may have effects on the prediction efficiency of the model. The ANN applied techniques could be an alternative to conventional linear regression model. ANN based models are constructed and adjusted by empirical training process. It is useful to solve and analyse the problems which could not be solved easily with traditional linear regression analysis. Especially, ANN-based modelling is useful to analyse multivariate nonlinear relationship [39]. For this reason, a higher prediction efficiency could be achieved by applying empirical training-based ANN modelling technique on predicting bioactivities of molecules [40]. In this study, ANFIS, an ANN-based system, was used to achieve high prediction efficiency of QSAR models. At first, the ANFIS applied QSAR models were developed using a data set from Müller et al. [4]. The predicted TEAC values by the developed QSAR models were presented in Table 3. Because carotenoids were limited to a variety of naturally occurred and commercially available ones, data sets with a relatively small sample size were available to be analysed. Bootstrap validation is an appropriate method to validate models with a small sample size [41,42]. Thus, in this study, MAE and q-square values of developed models were estimated by the bootstrap method. Both of the QSAR models with quantum chemical properties calculated by the PM6 and PM7 semi-empirical methods had high prediction efficiencies (Table 3 and Fig 3).

**Table 3:** Experimental and predicted TEAC of carotenoids.

	Experimental TEAC by Müller et al.[4]	Predicted TEAC	
		PM6	PM7
**α-Carotene**	3.3	2.79	2.83
**Antheraxanthin**	2.0	1.80	1.71
**Astaxanthin**	0.8	0.87	0.92
**β-Carotene**	3.1	3.15	3.21
**β-Cryptoxanthin**	3.2	2.97	3.01
**Canthaxanthin**	0.8	1.11	1.24
**Capsanthin**	2.0	1.63	1.51
**Echinenone**	2.2	2.10	2.11
**Lutein**	2.0	2.01	1.86
**Lycopene**	3.9	3.89	3.89
**Neoxanthin**	1.1	1.60	1.68
**Neurosporene**	2.1	2.55	2.51
**Phytoene**	1.0	1.00	1.00
**Phytofluene**	1.0	0.99	0.97
**Rubixanthin**	3.3	3.35	3.25
**Violaxanthin**	1.6	1.21	1.32
**Zeaxanthin**	1.9	2.30	2.34
**Mean absolute error**		0.22 ± 0.04	0.24 ± 0.05
**Q-square**		0.905 ± 0.043	0.895 ± 0.049

**Fig 4:**
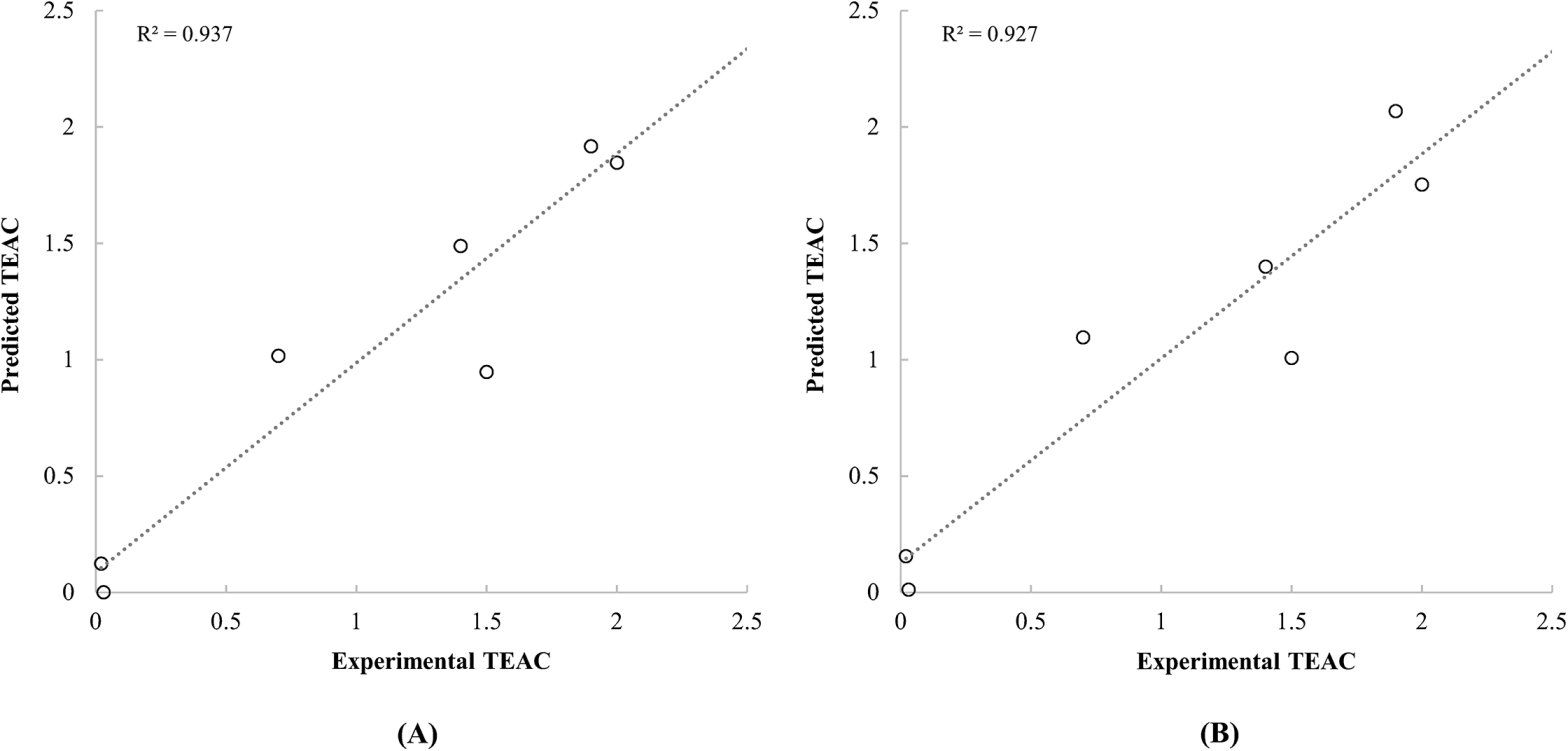
Scatter plot between the experimental TEAC of carotenoids reported by Miller et al. [5] and the predicted TEAC based on the quantum chemical descriptors calculated by PM6 (A) and PM7 (B) methods.

**Fig 5:**
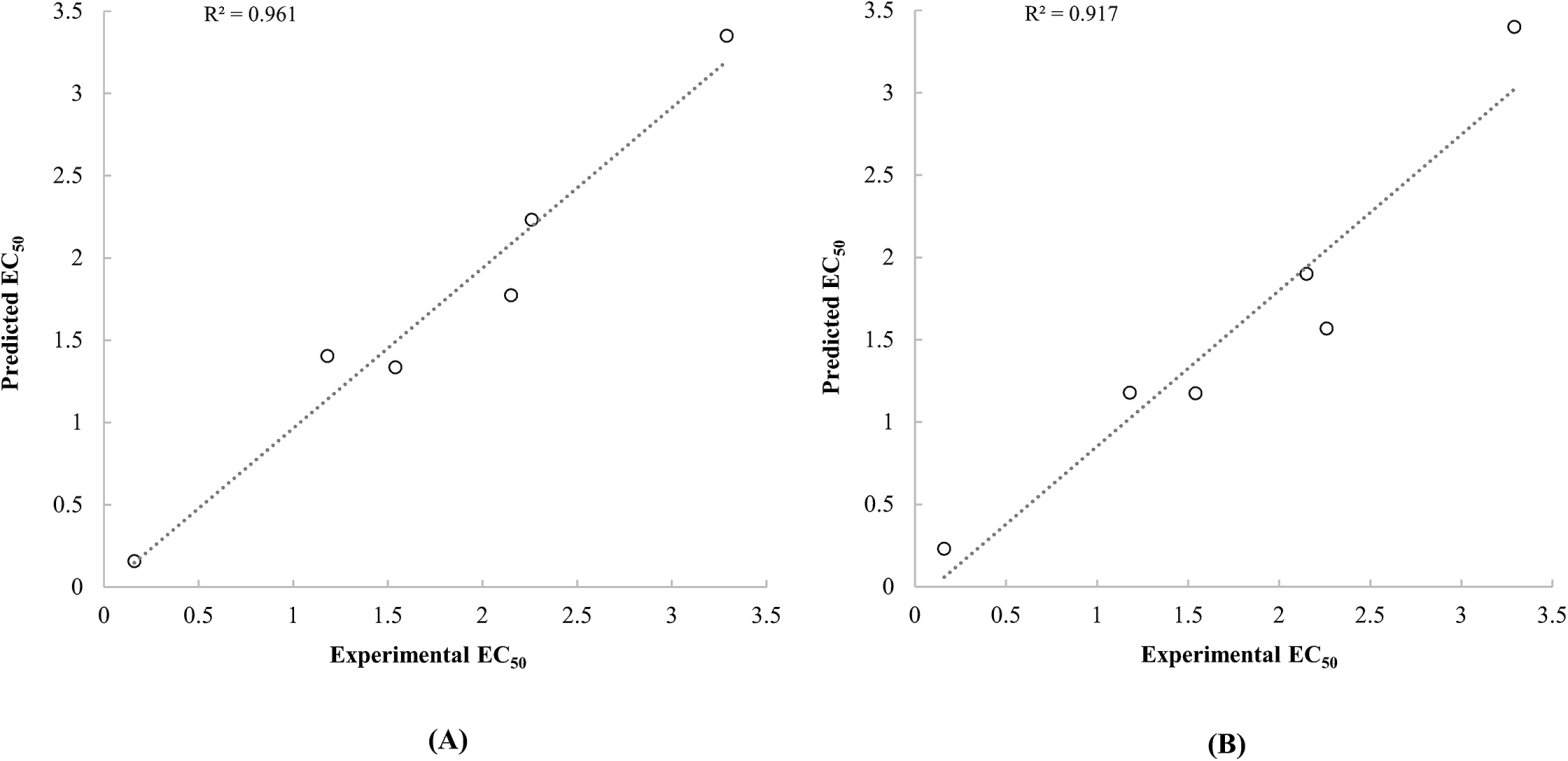
Scatter plot between the experimental EC50 of carotenoids reported by Jiménez-Escrig et al. [20] and the predicted TEAC based on the quantum chemical descriptors calculated by PM6 (A) and PM7 (B) methods.

To verify the reliabilities of the selected quantum chemical properties (i.e., ***I***, ***I***_***cat***_, and ***μ***_***cross***_) and the design of developed QSAR models, QSAR models were developed with radical scavenging activity data sets from other previous studies. The TEAC values of 8 carotenoid species were adapted from Miller et al. [5] and the EC50 data of 6 carotenoid species were adapted from Jiménez-Escrig et al. [28]. The results from correlation analysis of these data sets are presented in Table 4. The ***I***, ***I***_***cat***_, and ***μ***_***cross***_ of carotenoids calculated by both of the PM6 and PM7 were significantly correlated with the TEAC values from Miller et al. at p-value below 0.001. Although statistical significances were low on the correlation between experimental EC50 and quantum chemical properties of carotenoids due to a small sample size of the data set by Jiménez-Escrig et al. (n = 6), positive correlations were observed between EC50 and selected quantum chemical properties of carotenoids. A previous QSAR study regarding on radical scavenging activities of carotenoids by Soffers et al. [15] reported a positive relationship between the ionisation energies and the TEAC values using single data set constituted of 9 carotenoids. In addition to the previous study, this positive tendency was also verified using several number of data sets in this study. Kleinová et al. [16] also constructed a QSAR model for electrochemical redox potentials of carotenoids using polarizability as an independent variable. Since the polarizability was closely correlated with ionisation energy [43], the use of polarizability as an independent variable seemed adequate. However, based on electrochemical properties of carotenoids, the results from the previous study could not be directly interpreted to radical scavenging activities of carotenoids. In the present study, the reliabilities of selected quantum chemical properties for predicting radical scavenging activities and the prediction efficiency of ANFIS applied QSAR models were confirmed by applying various data sets (Figs 4 and 5).

**Table 4:** Pearson’s correlation coefficients between radical scavenging activities and quantum chemical descriptors of carotenoids calculated by PM6 and PM7 methods.

	TEAC by Miller et al. [5]		EC50 by Jiménez-Escrig et al. [28]	
	PM6	PM7	PM6	PM7
***I***	-0.923***	-0.917***	0.892*	0.850*
***I***_***cat***_	-0.937***	-0.942***	0.724	0.812*
***μ***_***cross***_	-0.892***	-0.914***	0.830*	0.802

*p < 0.05

**p < 0.01

***p < 0.001

***I***, ionisation energy of neutral carotenoid.

***I***_***cat***_, ionisation energy of cationic carotenoid.

***μ***_***cross***_, product of chemical potential of neutral and monovalent cationic carotenoid.

## Conclusions

Ionisation energies and chemical potentials of neutral and monovalent cationic carotenoid molecules were demonstrated as descriptors that describe the radical scavenging activities of carotenoids. Although some of the previous studies reported a significant relationship between quantum chemical descriptors and radical scavenging activities of phytochemicals [13,15,18], however, any report that analyses the radical scavenging activities of carotenoids quantitatively has not been found. In addition, in this study, molecular properties of cation molecules were calculated for QSAR models as well as neutral molecules. These characteristics could be used in computer-aided drug design (CADD) and cheminformatics areas for predicting and analysing molecular activities. Applying ANFIS on QSAR, high prediction efficiencies were also achieved. The results from this study suggest that the ANFIS could be a practical approach for developing QSAR models. Although the small sample sizes of data sets might weaken the significance of QSAR models, consistent tendencies, which were observed on various data sets, could demonstrate the reliabilities of selected quantum chemical descriptors and the significance of QSAR models.
